# Comparison of Three Domestications and Wild-Harvested Plants for Nutraceutical Properties and Sensory Profiles in Five Wild Edible Herbs: Is Domestication Possible?

**DOI:** 10.3390/foods9081065

**Published:** 2020-08-06

**Authors:** Costanza Ceccanti, Marco Landi, Luca Incrocci, Alberto Pardossi, Francesca Venturi, Isabella Taglieri, Giuseppe Ferroni, Lucia Guidi

**Affiliations:** 1 Department of Agriculture, Food and Environment, University of Pisa, 56124 Pisa, Italy; marco.landi@unipi.it (M.L.); luca.incrocci@unipi.it (L.I.); alberto.pardossi@unipi.it (A.P.); isabella.taglieri@for.unipi.it (I.T.); giuseppe.ferroni@unipi.it (G.F.); lucia.guidi@unipi.it (L.G.); 2 Interdepartmental Research Center Nutrafood “Nutraceuticals and Food for Health”, University of Pisa, 56124 Pisa, Italy

**Keywords:** wild edible herbs, soilless, pot, open field, nutraceutical profile, sensory profile

## Abstract

In this study, five wild edible herbs traditionally consumed in the Tuscany region (Italy) were evaluated for their potential in human nutrition. The nutraceutical characterization of *Rumex acetosa*, *Cichorium intybus*, *Picris hieracioides*, *Sanguisorba minor*, and *Plantago coronopus*, as well as their sensory profile were reported. Additionally, a preliminary assessment of completely different domestication of the wild species (named “soilless”, pot, and open field) was conducted to verify the possibility of their marketability, which is impossible if the plants are only gathered as wild. The open field domestication allowed to obtain plants with nutraceutical and sensory profiles similar to those of the wild species, especially in *C. intybus*, *P. hieracioides*, and *S. minor.* The pot domestication allow to obtain plants with chlorophyll and carotenoid contents close to those of the wild species, as well as a lower total phenolic and flavonoid content and ascorbic acid content than wild species. In the “soilless” method, *R. acetosa* and *P. coronopus* exhibited a high quality in terms of phytochemicals and antioxidant activity. Afterward, the sensory profile was strongly affected by the domestication in terms of the palatability, except for *R. acetosa* and *P. coronopus*, which displayed Hedonic Index (HI) values close to the consumer acceptability limit (HI = 6). A sensory profile similar to that of wild species was reported in open field domestication, whereas a worse sensory profile was reported in *P. hieracioides* and *C. intybus* domesticated using the soilless method. Finally, according to the preliminary assessment carried out in this study through an analysis of the general nutraceutical properties, *S. minor* was shown to be the most promising species thanks to its intrinsically highest nutraceutical properties considering the marketability of wild edible herbs as “new” functional food. However, further research on the bioavailability and bioactivity tests of nutraceutical compounds present in this species are required to confirm the findings of this study.

## 1. Introduction

In the last few decades, the interest in wild edible herbs has increased thanks to their already known medicinal properties [1,2,3,4], as well as their use in dietary supplements as a source of nutraceutical properties and bioactive compounds [5,6,7,8]. This is due to the high content of phytochemicals found in many wild edible species, which makes them useful as a “new” functional food. A functional food can be defined as “a food that provides medical or health benefits, including the prevention and/or treatment of a human disease” thanks to the content of phytochemicals, mainly synthesized by shikimic and phenylpropanoid pathways, as well as the terpenoid pathway [9], even though the consumed amounts of wild edible herbs cannot currently be considered sufficient to ensure that these herbs achieve a bio-functionality in the human diet. However, some of these herbs have already been characterized for uses other than as food, such as pharmaceutical and medicinal ones. For example, Petropoulos et al. [10] isolated 5-*O*-caffeoylquinic acid, caftaric acid, cichoric acid, kaempferol 3-glucuronide, and quercetin 3-*O*-β-d-glucuronide from *Cichorium spinosum* L. leaves and Mikropoulou et al. [11] found predominantly phenolic acids, such as 3-caffeoylquinic acid, 5-caffeoylquinic acid, cichoric acid, and caftaric acid, and flavonoid derivatives, such as quercetin and luteolin glucuronides, in *C. intybus* leaves. The final aim of such studies was the characterization of phytochemicals contained in wild and domesticated edible herbs and their role in preventing human diseases [12,13,14]. In a world where the average age in developed countries has lengthened, age-related diseases have obviously increased in number [15]. It is well-known that phytochemicals typical of some wild edible herbs have shown potential roles in the prevention of age-related diseases; for example, β-carotene and vitamin A have been considered useful in the treatment of age-related eye diseases, such as cataracts, glaucoma, and macular degeneration [16]; vitamin C, caffeoylquinic acids, phenolic acids, and flavonoids have been reported to display high antioxidant and anti-inflammatory activity and have consequently been proven to be useful in the prevention of inflammatory conditions, such as pulmonary disease, rheumatoid arthritis, and renal disease [17,18]; glucosynolates have exhibited antiproliferative activity against colon cancer cells [19]; and stilbenes have been considered useful in the prevention of cardiovascular and cerebrovascular disease, coronary heart disease, and hypertension, thanks to their capacity to inhibit the oxidation of low-density lipoproteins [20]. Nevertheless, in terms of the sensory profile, the main acquiring and repeated acquiring criteria persist [21]. For all of these reasons, researching the efficient domestication of wild edible herbs able to maintain their nutraceutical properties and palatability for consumers and making the commercialization of these herbs as a “new” functional food possible is now a requirement for ensuring their marketability [22]. In this study, the domestication of five wild species [sorrel (*Rumex acetosa* L.), chicory (*C. intybus*), hawkweed oxtongue (*Picris hieracioides* L.), salad burnet (*Sanguisorba minor* Scop.), and buck’s horn plantain (*Plantago coronopus* L.)] was evaluated to investigate the opportunity to grow “new” horticultural crops and to extend the offer of food markets [23]. These species are typical wild edible herbs of the Tuscany region, which is a territory characterized by different vegetation belts from sea level to the highest peak of Monte Prado (about 2000 m a.s.l.), representing a typical landscape of a Mediterranean area.

Sorrel (*R. acetosa*) is a perennial herb belonging to the Polygonaceae family. This species is a common plant in grassland habitats, mainly distributed in Europe, Asia, Africa, and North America, predominantly in the northern hemisphere [24]. Sorrel possesses long and rhizomatous roots and the leaves are fresh, green, and constitute a basal rosette [25]. The leaves are used as fresh or cooked vegetables in many cultures around the world [25]. The rosette contains vitamin C, flavonoids, phenolic acids, and proanthocyanidins with important pharmacology activity (antiproliferative and antioxidant activity and discomfort and distress stomach treatments) [24]. The main phenolic acids found in sorrel are gallic acid, ellagic acid, protocatechuic acid, ferulic acid, *p*-cumaric acid, rosmarinic acid, vanillic acid, and sinapic acid and they are characterized by antioxidant and anti-inflammatory activity, whilst the main proanthocyanidins are epicatechin and epicatechin derivatives with confirmed antiviral activity [24].

Chicory (*C. intybus*) is a perennial herb belonging to the Asteraceae family, which can tolerate extreme temperatures during both vegetative and reproductive growth. In fact, it is a widespread weed all over the world, with a cosmopolitan distribution, and can be found in roadside, pasture, waste ground, and other humanized environments [25]. The taproot contains up to 40% of inulin, which is a fructose polymer that has a slight impact on blood sugar and is thus suitable for diabetics [26]. The rosette, composed by basal lanceolate leaves [25], is commonly used in cooking as fresh or cooked vegetables, even though this species is more known for its various functions in the human body thanks to its phytochemical content [18]. Important phytochemicals have already been found in chicory leaves, such as phenols (especially caffeoylquinic acids and flavanol derivatives [18,27]), vitamin A and C, and chicoric acid, which can stimulate the immune system and prevent inflammation and bacterial infections [28]. Among the five examined edible herbs of this study, the chicory is the one whose domestication is already widespread, mainly in Northwestern Europe, India, South Africa, and Chile, in order to produce the chicory taproot as a coffee substitute or for inulin extraction [18].

Hawkneed oxtongue (*P. hieracioides*) is a biennial or short-lived perennial herb belonging to the Asteraceae family. It is widespread across Europe and has been introduced in North America, Southern Africa, and Australia [29]. This species is characterized by a fasciculate root and a rosette of basal lanceolate leaves, common in the Asteraceae family [30]. In the last decades of the twentieth century, the attractiveness of this species was due to the discovery of numerous terpenoid glycosides (especially sesquiterpene glycosides, such as picriosides A and B and picroniosides A and B) in the methanolic extract of the air-dried whole plant [31,32]. The use of sesquiterpene lactones in pharmacopeia is well-known for fever treatment and other inflammation treatment [31,32], but the medicinal properties of the hawkneed oxtongue are still unknown. The rediscovery of this species is due to the renewed interest in traditional cooking [33] and, for this reason, the effect of this species on human health as a hypothetic functional food should be determined.

Salad burnet (*S. minor*) is a perennial herb belonging to the Rosaceae family and its diffusion has been shown in the Sinai Peninsula, Egypt, as well as in temperate areas in Europe, especially in grassland and shrublands [34]. This species is characterized by stems ranging from 2 to 70 cm in height in drought and moist environments, respectively, containing pinnately basal leaves and a stout taproot which has a high water-storing capacity, allowing the drought resistance. The stems with leaves are conformed in a basal rosette [35]. Salad burnet’s leaves are well-known for medicinal properties, hypoglycaemic activity, and anti-inflammatory activity, due to high levels of α-tocopherols, β-carotene, and vitamin C and E [36]. Moreover, many antioxidant compounds have been reported by the literature, such as benzoic acids (gallic and ellagic acid and glycosidic derivatives), flavanols (kaempferol and quercetin), and flavones (apigenin, baicalein, and 7,3’,4’-trihydroxyflavone) [34,36].

Buck’s horn plantain (*P. coronopus*) is a perennial herb belonging to the Plantaginaceae family. This edible herb possesses rhizomatous roots and its leaves are elongated and arranged in a basal rosette [37]. It is used in mixed salad for its mild, nutty flavor and crunchy texture, since it has always been appreciated for its salty taste and high nutritional value typical of this species [38]. In fact, the leaves display a high content of phenolic compounds, as well as amino acids (serine, proline, arginine, leucine, glycine, lysine, and threonine), including essential amino acids such as phenylalanine and tyrosine, precursors of the phenylpropanoid pathway, and minerals (calcium, magnesium, sodium, potassium, etc.) [39,40]. The domestication of this species has never been carried out, even though its salt tolerance is well-known in the literature [38].

Despite the high ethnobotanical biodiversity of these species, their common widespread diffusion in different environments (with different temperatures, soils, moisture, irradiation, etc.) suggests a high adaptability. For this reason, the maintenance of wild nutraceutical properties able to allow the commercialization of these species as “new” functional food can be achieved through the adopted domestication.

Despite the common use of the five wild edible herbs in traditional Mediterranean recipes and their diffusion in the Tuscany region and the whole Mediterranean area, their sensory profile is unknown and studies on their sensory and organoleptic characteristics were not found by the authors.

The aim of this work was to conduct a preliminary characterization of the sensory and phytochemical profiles of five wild edible herbs, to identify their potential nutraceutical properties and the sensory profile, and to determine the range of palatability and marketability of these species in terms of “new” functional food. Preliminary research on different domestications able to efficiently maintain the phytochemical properties and sensory profile of the examined wild edible herbs is another step of this study. The authors are aware that the domestications analyzed in this study had multiple differences in terms of the pedo-climatic conditions, which could not be controlled by our experiment, and that the number of biological replicates and the consideration of only one harvest season was limited in a scientific way; however, they want the present study to be interpreted as a preliminary and general study to be explored in future research. Therefore, the aim of this work is to test whether consolidated practices employed by local farmers for the production of widely used vegetables can be efficiently applied for wild species domestication, in view of a production upscale of those wild herbs. Although preliminary, this dataset, dealing with the general phytochemical characterization of five edible herbs grown with three different cultivation techniques and compared to harvesting plants in the wild, can generate knowledge, since limited efforts on the domestication of these species are present in the literature [40]. Accordingly, this work may contribute to increasing knowledge about the sensory profile, phytochemicals, and nutraceutical properties of wild edible herbs, leading to the recognition of them as “new” functional food.

## 2. Materials and Methods

### 2.1. Plant Materials, Growth, and Experimental Conditions

Ten seedlings of *R. acetosa*, *C. intybus*, *P. hieracioides*, *S. minor*, and *P. coronopus* were selected and labeled after their emergence in the Pisa area, in the Tuscany region (Italy), during the spring of the year 2019. The plants were gathered as wild (W) specimens after one month of the wild birth of the plant.

Additionally, the vegetable material of each species under investigation was provided by a fruit and vegetable wholesaler (Tirrenofruit S.r.l., Florence, Italy), who collects the products from local farmers. These farmers cultivate their product following consolidated practices for the production of widely used crops. In this study, these practices are referred to as “soilless” (SS), pot (P), and open field (OF). In this investigation, they simply transferred those methodologies to our wild edible species. Details of the three domestications and the wild harvest are reported in Table S1.

All of the species, wild and domesticated, were collected before flowering, when the length of leaves was approximately 5–10 cm, paying attention to ensure the collection of the youngest leaves in the basal rosette of *C. intybus*, *P. hieracioides*, *P. coronopus*, and *R. acetosa* and the youngest stems of the basal rosette of *S. minor.* In fact, the collection of the youngest aerial part of the species is a commercial choice since, in traditional cooking, the consumption of the tender young leaves and stems is well-documented [12,18,25]. After the growth period, plants were cut up-right above the substrate or the soil level and sampled. Plant material provided from ten wild plants for each species or ten cultivated plants for each species for each domestication was pooled and randomly sampled in three replicates of 50 g. Part of the pooled sampled material from the wild-collection and from the different domestications was analysed immediately for the sensory profile. Another part of it was then processed as fresh material, frozen in liquid nitrogen, and left at −80 °C until the biochemical analyses.

### 2.2. Determination of the Total Phenolic and Flavonoid Content

Fresh stored material (1 g) was homogenized with 4 mL 80% (*v/v*) methanol solution and then sonicated for 30 min at 4 °C. Samples were centrifuged at 10,000 *g* for 15 min at 4 °C and supernatants (2 mL) were picked up, centrifuged for 3 min at 7000 *g*, and used for the analysis. The total phenolic (TP) content was determined by the Folin-Ciocalteu (FC) method described by Dewanto et al. [41], with minor modifications, using the Ultrospec 2100 Pro spectrophotometer (GE Healthcare Ltd., Little Chalfont, UK). For the analysis, 10 μL of the sample was placed in a cuvette. Then, 125 μL of the FC reagent and 115 μL of distilled water were added to each sample. The reaction was incubated at 25 °C for 6 min. Then, 1.25 mL 7% (*w/v*) Na_2_CO_3_ solution was added and samples were incubated for 90 min in dark conditions. Immediately, the absorbance was measured at 760 nm. The TP content was expressed as milligram equivalents of gallic acid per g of fresh weight (mg GAE g^−1^ fresh weight, FW).

The total flavonoid (TF) content was spectrophotometrically determined using the AlCl_3_ complexation method described by Du et al. [42], with some modifications. Each sample (100 μL) was mixed with 30 μL 5% (*w/v*) NaNO_2_ solution and 440 μL deionized water. The reaction was incubated at 25 °C for 6 min. A total of 30 μL 10% (*w/v*) AlCl_3_ was added and the mixture was incubated for another 6 min. Then, 400 μL 4% (*w/v*) NaOH solution was added. The absorbance of the mixture at 415 nm was measured after 15 min of reaction. The total flavonoid content was expressed as mg catechin equivalents (CAE) per g FW.

### 2.3. Chlorophyll and Carotenoid Analysis

Chlorophyll and carotenoid analysis was performed following the method described by Porra et al. [43], with minor modifications. Fresh stored material (0.15 g) was extracted and homogenized in 10 mL 80% (*v/v*) acetone and agitated in the dark for 4 days. The total chlorophyll content was determined by summarizing chlorophyll *a* (determined by the increase in absorbance at 663 nm) and chlorophyll *b* (determined by the increase in absorbance at 648 nm). The carotenoid content was determined by the increase in absorbance at 470 nm. The total chlorophyll and carotenoid contents were expressed as mg per g FW.

### 2.4. Ascorbic Acid Assay

The ascorbic acid amount was measured using a spectrophotometric method [44]. The extraction was obtained by homogenizing fresh material (0.3 g) in 1 mL 0.6% (*w/v*) trichloroacetic acid and centrifuging it at 10,000 *g* for 10 min. A solution of 100 μL sample, 50 μL 10 mM dithiothreitol, and 100 μL 200 mM sodium-phosphate buffer was mixed and incubated at 42 °C for 15 min. During this period, the dehydroascorbate was reduced to ascorbate. After 15 min, 50 μL 0.5% (*w/v*) N-ethylmaleimide, 250 μL 10% (*v/v*) trichloroacetic acid, 200 μL 42% (*w/v*) orthophosphoric acid, 200 μL 4% (*w/v*) 2,2’-dipyridyl (diluted in 70% (*v/v*) ethanol solution), and 100 μL 3% (*w/v*) FeCl_3_ were added to the sample. The mixture was incubated at 42 °C. The absorbance of the mixture at 525 nm was measured after 40 min of reaction. The ascorbic acid content was expressed as mg ascorbic acid (ASA) per g FW.

### 2.5. 2,2-Diphenyl-1-picrylhydrazyl Hydrate (DPPH) Free Radical Scavenging Assay

The DPPH free radical scavenging capacity of each sample was determined according to the method described by Brand-Williams et al. [45]. Briefly, a 3.12 × 10^−5^ M DPPH solution diluted in methanol was prepared. For the assay, the same extract of TP and TF content was utilized. A total of 10 μL of an extract (with appropriate dilution, if necessary) was added to 990 μL of methanolic DPPH solution. The change in absorbance at 515 nm was measured after 30 min of incubation. The in vitro antioxidant capacity based on the DPPH free radical scavenging ability of the extract was expressed as mg Trolox equivalents (TEAC) per g FW.

### 2.6. Sensory Profile

Sensory profiles of the analyzed herbs were determined by a descriptive analysis by a panel of trained assessors (10 assessors, 6 females and 4 males, aged between 23 and 60 years). All of the involved assessors were included in the “expert panel” of the Department of Agriculture, Food and Environment (DAFE) of University of Pisa and the DAFE internal procedure for assessor selection and the training was applied as reported in previous papers [46,47].

**Figure 1:**
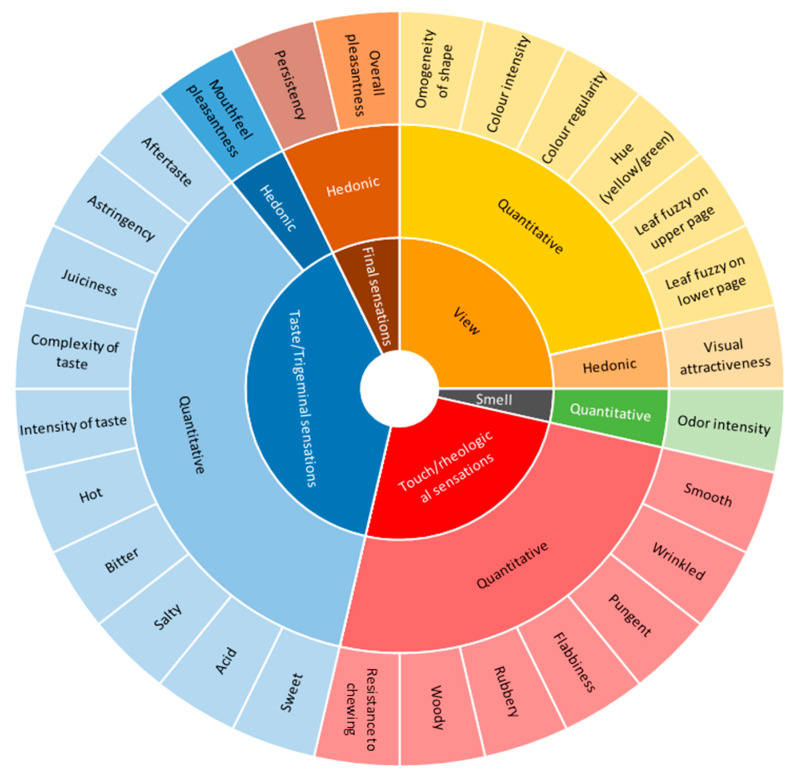
Sensory wheel specifically developed to separately describe each wild-collected and domesticated species using three different methods: “soilless”, pot, and open field.

Starting from this general protocol, a specific training session was organized for all of the selected panelists before the start of the specific tasting sessions. The aim of this specific training session was to design the specific method of the sensory evaluation of wild edible herbs and all of the trained panelists were also involved in a consensus panel specifically aimed at generating descriptors and their definitions [48]. A final set of 28 descriptive parameters, including both quantitative and hedonic attributes, was individuated by agreement among panelists and an innovative sensory wheel specific for the tasting of wild edible herbs was developed (Figure 1).

The panelists always had the option to include relevant specific observations for each analysed herb in the “others” parameter.

All of the sessions were conducted in the morning, in a well-ventilated, quiet room and in a relaxed atmosphere. For each tasting session, three to five leaves were provided to each panelist, avoiding any indication about the domestication utilized, and the samples related to the same plant treated differently were assessed separately during the same morning. The samples were presented in a different order in each tasting session and 10-min intervals were allowed between each sample; they were randomly labeled with a three-digit numeric code and provided to assessors in a double-blind presentation to avoid any expectation error. Furthermore, a sample for each species was randomly replicated to verify the performance of the panel in each tasting session. For evaluation, each assessor was provided with filtered water and asked to cleanse their palate between tastings.

To avoid cross contamination, all leaves were eliminated from the room before the start of a new tasting session and the different plant species were assessed by the same group of panelists, but during different mornings.

The panelists rated the intensity of each parameter (Figure 1) from 0 (minimum scale) to 9 (maximum scale), including visual, aroma, and taste/touch attributes, as well as some hedonic parameters, in order to provide indications about the whole quality of the tasted herbs.

The overall organoleptic quality of the different herbs was expressed as Hedonic Index (HI), calculated on the average values attributed during panel tests to each hedonic parameter (Visual attractiveness, Mouthfeel pleasantness, Persistency, Overall pleasantness—see Figure 1); the obtained values were then reported in a scale 0–10 as follows:

HI= Average [Hedonic indexes] × 1.11(1)

### 2.7. Statistical Analysis

The nutraceutical analyses were carried out in triplicate and the results are presented as the mean ± standard deviation (SD). One-way analysis of variance (ANOVA) with Fisher’s least significant difference (LSD) post-hoc test (*p* = 0.05) was carried out using GraphPad (GraphPad, La Jolla, CA, USA). The source of variability is represented by wild-collection and the different domestications, with the sole purpose of verifying the maintenance of wild nutraceutical properties after preliminary attempts of different domestication methods.

Sensory analysis results were processed by Big Sensory Soft 2.0 (ver. 2018). Sensory data were analysed by two-way ANOVA, with panelists and wild or domesticated specimens as sources of variability [49].

Pearson’s coefficient was calculated in order to define the correlation among quantitative and hedonic parameters, using XLSTAT version 1 April 2019 (Addinsoft Inc. 244 Fifth Avenue, Suite E100, New York, NY 10001, USA).

## 3. Results

### 3.1. Total Phenolic (TP) and Total Flavonoid (TF) Content

**Figure 2:**
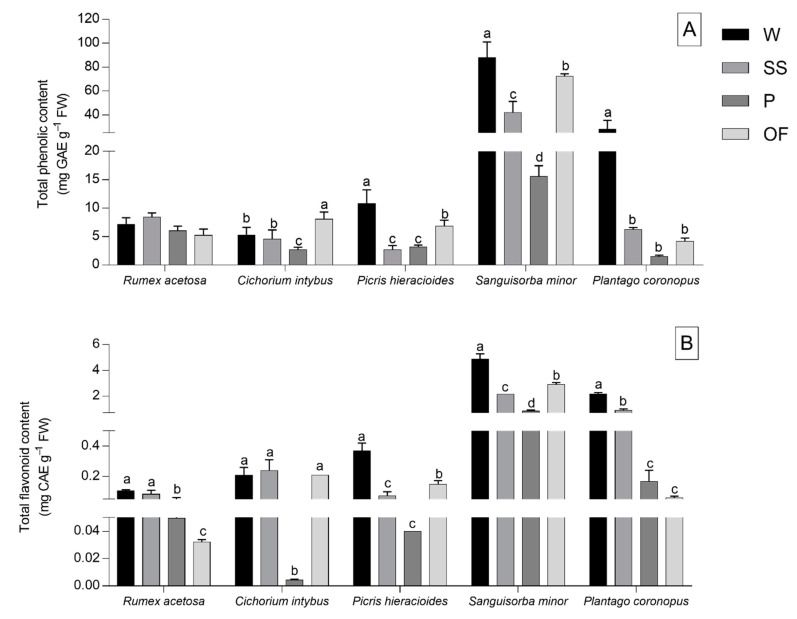
Total phenolic (**A**) and total flavonoid (**B**) contents of *Rumex acetosa*, *Cichorium intybus*, *Picris hieracioides*, *Sanguisorba minor*, and *Plantago coronopus* wild-collected (W) and domesticated according to three different methods: “soilless” (SS), pot (P), and open field (OF). Each value is the mean (±SD) of three replicates. Means keyed with a different letter are significantly different for *p* = 0.05 considering each species independently and following one-way ANOVA, with wild-collection or the different domestications as the variability factor.

The TP content results are shown in Figure 2A. *R. acetosa* leaves exhibited no differences in the TP content among domestications and wild collected plants. Additionally, the TP content displayed higher results in wild *P. coronopus*, *S. minor*, and *P. hieracioides* plants if compared with all domestications, even though the OF domestication exhibited the results closest to those of wild plants (Figure 2A).

Moreover, wild *C. intybus* displayed a TP content similar to that of SS domesticated leaves and leaves of plants domesticated with OF method demonstrated a slightly higher TP content if compared with the wild leaves.

The pattern of the TF content was very close to that of the TP content, especially for *P. coronopus* and *S. minor* (Figure 2B) and the highest TF values were reported in leaves of wild-collected species (2.2 and 4.8 mg CAE g^−1^ FW, respectively). In *R. acetosa*, the SS domestication exhibited similar results to wild collection. Leaves of plants domesticated with SS and OF methods showed a TF content close to the TF content of wild *C. intybus* leaves, whilst in *S. minor*, *P. hieracioides*, and *P. coronopus*, all of the domestications displayed a lower TF content than that of wild plants. However, leaves of plants domesticated with OF method exhibited the results closest to those of wild collection in *P. hieracioides* and *S. minor* and SS domestication in *P. coronopus.*

### 3.2. Chlorophyll and Carotenoid Content

**Figure 3:**
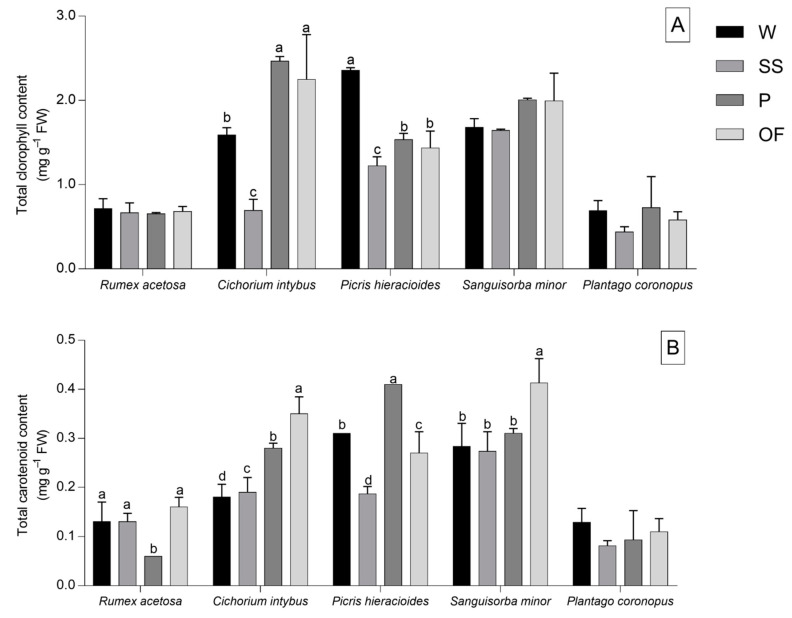
Total chlorophyll (**A**) and carotenoid (**B**) contents of *Rumex acetosa*, *Cichorium intybus*, *Picris hieracioides*, *Sanguisorba minor*, and *Plantago coronopus* wild-collected (W) and domesticated according to three different methods: “soilless” (SS), pot (P), and open field (OF). Each value is the mean (±SD) of three replicates. Means keyed with a different letter are significantly different and means not keyed with letters are not significantly different for *p* = 0.05, considering each species independently and following one-way ANOVA, with wild-collection or the different domestications as the variability factor.

Figure 3A shows that each species under investigation contained similar concentrations of total chlorophylls between wild and different domestications, except for *C. intybus* and *P. hieracioides.* In fact, all domestications reported a lower chlorophyll content if compared with wild *P. hieracioides* leaves, whereas leaves of plants domesticated with OF and P methods demonstrated a higher chlorophyll content than wild *C. intybus* leaves (Figure 3A).

On the contrary, Figure 3B shows that each wild-collected and domesticated species showed distinct levels of carotenoids, except for *P. coronopus*. In *R. acetosa*, leaves of plants domesticated with SS and P methods had a carotenoid content close to that of wild leaves. In *C. intybus*, all domestication methods allow to obtain plants with a higher carotenoid content than the wild leaves, whereas in *S. minor*, leaves of plants domesticated with SS and P methods displayed similar carotenoid contents to wild plants. The P method allow to obtain plants with a higher carotenoid content than that of wild *P. hieracioides* leaves, whilst the other methods reported lower results when compared with the wild leaves of the same species.

### 3.3. Ascorbic Acid and DPPH Free Radical Scavenging

**Figure 4:**
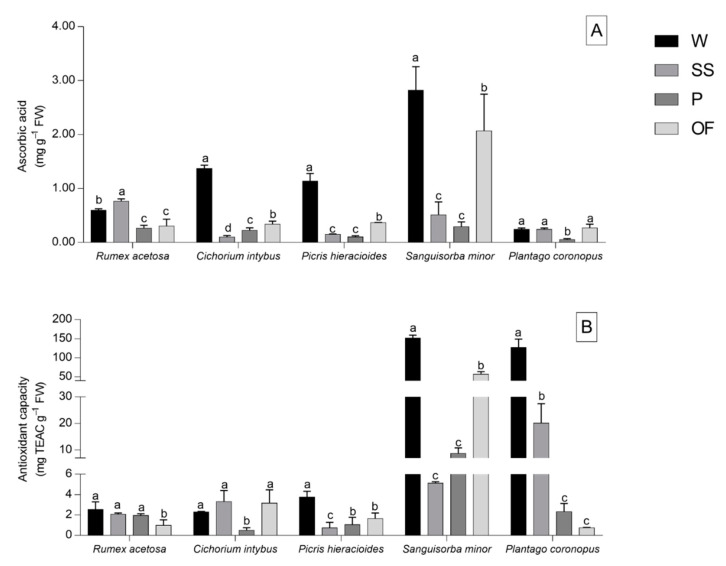
Total ascorbic acid content (**A**) and antioxidant capacity (**B**) of *Rumex acetosa*, *Cichorium intybus*, *Picris hieracioides*, *Sanguisorba minor*, and *Plantago coronopus* wild-collected (W) and domesticated according to three different methods: “soilless” (SS), pot (P), and open field (OF). Each value is the mean (±SD) of three replicates. Means keyed with a different letter are significantly different and means not keyed with letters are not significantly different for *p* = 0.05, considering each species independently and following one-way ANOVA, with wild-collection or the different domestications as the variability factor.

The ascorbic acid content and the antioxidant capacity of fresh plant extracts of the analysed species are shown in Figure 4.

Wild-collected species exhibited the highest ascorbic acid content in *C. intybus*, *P. hieracioides*, and *S. minor* (Figure 4A). In all three species, the domestication resulted in a decrease in the ascorbic acid content, even though leaves of plants domesticated with OF method reported an ascorbic acid content closer to that of the wild collection. *P. coronopus* leaves displayed similar results to the wild leaves domesticated with SS and OF methods, whereas *R. acetosa* only demonstrated such values for SS domestication (Figure 4A).

The total antioxidant capacity was determined by a DPPH assay. *R. acetosa* leaves displayed similar antioxidant activity to wild leaves when domesticated using SS and P methods, and *C. intybus* leaves when domesticated using SS and OF methods (Figure 4B). In *P. hieracioides* and *S. minor* plants, the wild collection reported the highest antioxidant activity and the leaves of plants domesticated with OF method reported results closer to those of wild *S. minor* plants, whilst leaves of plants domesticated with SS method displayed antioxidant activity closer to that of wild *P. coronopus* leaves (Figure 4B).

### 3.4. Sensory Profile

Based on the two-way ANOVA test with the panelists and domestications as the main effects, most of the evaluated quantitative and hedonic parameters for each analysed species were significantly different and the wild species exhibited the highest results for most of the evaluated parameters in all of the examined species (Table 1, Table 2, Table 3, Table 4 and Table 5).

Therefore, the sensory profile of the wild analysed species was differently influenced by the domestications (Table 1, Table 2, Table 3, Table 4 and Table 5) and the differences among wild and each domesticated species cannot be reduced to a general trend. The visual attributes and the touch/rheological sensations varied significantly with the domestications for all of the species, with the only exception of *S. minor*. Conversely, the taste attributes and the trigeminal sensations appeared to be less influenced by the domestication.

The Pearson’s correlation coefficient among quantitative and hedonic parameters was determined for each species and data are reported in Table S2.

The sensory profile of *R. acetosa* was significantly influenced by few attributes: the homogeneity of dimensions and the wrinkled shape of leaves were evaluated as positive attributes, whilst the presence of a fuzzy underside on the leaves, a salty taste, and a hot sensation were evaluated as negative attributes by panelists (Table S2). Comparatively, a great number of strong correlations among quantitative and hedonic parameters were individuated in the sensory profile of *C. intybus*, *P. hieracioides*, and *P. coronopus* (Table S2). For these species, a cross-analysis of the data reported by the two-way ANOVA test and the Pearson’s correlation test allowed us to underline the specific interaction among the wild collection and each method and the quantitative/hedonic parameters of each species under investigation. For example, an increase in hedonic parameters was found in *C. intybus* and *P. coronopus* domesticated in OF, followed by plants domesticated with SS method. Conversely, the quantitative parameters in the sensory profile of *P. coronopus* were differently influenced by the wild collection, as well as by the different methods, and, for this reason, clear individuation of the maintenance of wild palatability was very difficult.

**Figure 5:**
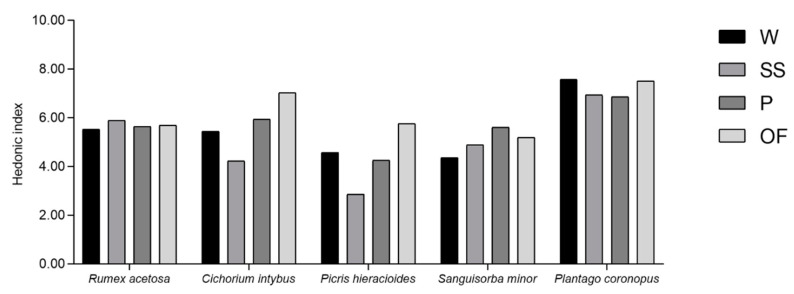
Hedonic index of *Rumex acetosa*, *Cichorium intybus*, *Picris hieracioides*, *Sanguisorba minor*, and *Plantago coronopus* wild-collected (W) and cultivated according to three different techniques: “soilless” (SS), pot (P), and open field (OF).

Finally, a qualitative Hedonic Index (HI) was evaluated, starting from the mean values attributed by panelists to the hedonic parameters, in order to verify the impact of the wild-collection and the different domestications on the sensory profile and, subsequently, on the consumer acceptability of the species under investigation (Figure 5).

According to the already observed results, the HI was strongly affected by the domestication for each species, except for *R. acetosa*, followed by *P. coronopus*, which showed an HI close to the consumer acceptability limit (HI = 6), regardless of the wild-collection or domestication type.

## 4. Discussion

As expected, most of the examined species showed the highest TP, TF, ascorbic acid content, and total antioxidant capacity in plants collected in the wild. The antioxidant capacity is affected by the phytochemical content; in fact, phytochemicals, including flavonoids and ascorbic acid, have been shown to be potent quenchers of Reactive Oxygen Species (ROS), such as ROO^•^, O_2_^•–^, H_2_O_2_, OH^•^, and *O_2_ and, consequently, they have been shown to have protective roles against inflammation and carcinogenesis [50]. Recent studies reported that the differences between wild and domesticated species are attributable to different genotypes and different domestications [51,52,53]. Gutiérrez-Velázquez et al. [51] showed a lower TP content in wild than domesticated watercress plants. Accordingly, some investigations conducted on wild and domesticated *C. intybus* reported a lower TP content in wild leaves than in domesticated ones [54,55,56], whilst Heimler et al. [57] reported a higher TP content in wild chicory. Epigenetic modifications in wild edible herbs may be the key factors modulating the expression of genes involved in the synthesis of phytochemicals [51]. At the same time, genetic replication results are difficult to obtain because of the gathering and collection of the young aerial part and its edibility before the flowering period and, in this way, the lack of the seed availability of the same plants collected as wild [58]. However, there has been an increase in evidence showing that wild edible herbs contain higher quantities of phytochemicals when compared to domesticated vegetables [59,60]. In this way, it seems that agronomic selection of domesticated and commercial cultivars has led to a decrease in the amount and variability of phytochemicals compared to those of wild edible herbs [52]. Therefore, wild edible herbs have been considered promising natural sources of potential genes for cultivated crop improvement due to their higher genetic variability and propagation in a large range of habitats without human selection [53]. In this study, different domestications were adopted to test the possibility to obtain reproducible and marketable products, paying particular attention to the maintenance of the nutraceutical properties of wild plants. In the comparison of wild harvested material and the domestication of the five wild edible species under investigation with OF method, it can be highlighted that this method allowed plants with TP, TF, and ascorbic acid content values close to those measured in wild plants of *C. intybus, P. hieracioides*, and *S. minor* to be obtained. The high solar radiation provided to OF-grown plants was a positive driver for the accumulation of phytochemicals, which may promote plant defense by possible oxidative stress induced by high irradiation [61]. Among solar wavebands, UV strongly promotes phytochemical compounds devoted to light protection, since UV-B radiation activates enzymes as phenylalanine amino lyase (PAL), a precursor enzyme of the phenylpropanoid pathway, and chalcone synthase, a precursor enzyme of flavonoid synthesis, thereby leading to the accumulation of phenolic compounds in plants [62,63]. Moreover, the higher temperature and lower humidity reported in OF method may also have affected the phytochemical accumulation. In fact, Wang and Zheng [50] reported that a high growth temperature significantly enhanced the TF content, consequently increasing the % inhibition of ROS in strawberries [50]. Moreover, research dealing with the effect of drought has shown that the limited water availability also provokes the accumulation of phytochemicals in plants [64,65].

The second preliminary attempt of domestication (P) allowed us to obtain plants with high chlorophyll and carotenoid contents, especially in *C. intybus*, *P. hieracioides*, and *S. minor*, whose levels were close to those of the wild species. Their more protected growing environment in comparison to that of the wild species could be the factor influencing the chlorophyll and carotenoid content. In fact, previous studies have shown that shade-grown leaves produced supplementary chlorophylls to capture diffuse radiation to be tunneled to photosynthesis [66] and that a high light intensity induces degradation of the chlorophyll content rate [67,68]. Conversely, P-grown plants accounted for lower levels of TP, TF, and ascorbic acid content and antioxidant activity than wild species. This could mainly be due to the lower irradiance experienced by greenhouse-grown plants [61], since the temperature and relative humidity of this method were close to those experienced by plants collected in the wild. Moreover, the use of peat as a substrate and nutrient fertirrigation provided optimal growth conditions, thereby not promoting activation of the secondary metabolism, usually devoted to protecting the plant against environmental cues and a limited availability of resources [61].

Finally, the last attempt of domestication (SS) allowed us to obtain plants with similar antioxidant activity in *R. acetosa*, *C. intybus*, and *P. coronopus* and similar ascorbic acid contents in *R. acetosa* and *P. coronopus*, when compared with results observed in the counterpart wild-grown plants. This domestication was characterized by a low temperature that could affect the phytochemical results. Schonhof et al. [69] showed that low temperatures increased the ascorbic acid content in broccoli because of the effect of a low temperature on ascorbic acid, which acts by limiting the utilization of excitation energy by photosynthetic carbon dioxide fixation [69]. Moreover, the *R. acetosa*, *C. intybus*, and *P. coronopus* patterns were in line with recent research comparing the effect of soilless and soil medium on the quality of plants [60,70]. Okonwu et al. [70] showed that the soilless medium improved the TF content in *Telfairia occidentalis* and Di Gioia et al. [60] reported that the total glucosynolate content in different varieties of rocket was significantly higher in soilless system than conventional soil system grown plants, suggesting that soilless systems can provide plants with a similar, or even higher, nutritional quality than conventionally grown plants [60,70].

Moreover, the results of this study confirmed the richness of TP, TF, and ascorbic acid content of *S. minor* independent of the domestication or wild harvest, as already reported by Karkanis et al. [71] and Ceccanti et al. [34], probably due to intrinsic factors of this species, and deep studies on this plant species are required to improve the knowledge on this promising herb as functional food. However, further research on the bioactivity and bioavailability of the bioactive compounds present in this species are needed to confirm or deny the findings of the present study. Additionally, the ascorbic acid values found in *C. intybus*, *P. hieracioides*, and *S. minor* were very similar to those found in common vegetables, such as broccoli and spinach, known to be high sources of ascorbic acid [72,73], whilst the range of the antioxidant capacity of *C. intybus* was higher than that reported by Llorach et al. [74] and that reported in common vegetables, such as lettuce and iceberg. A comparison of common vegetables is necessary to show the impact of the introduction of new functional food comprised of a mix of wild edible herbs could have on the human diet, replacing common salads. Moreover, the wild-collected and domesticated species of this study were characterized by a moderate content of chlorophylls, lower than that found in garden rocket, chicory cv. ‘Anivip’, wild rocket, dandelion, and chicory cv. ‘Monivip’ [75], as well as kale [76], and similar to that found in spinach [77]. Conversely, the carotenoid content was higher than that found in common leafy vegetables such as lettuce [78,79] and close to that found in baby spinach [80]. Finally, during the evaluation of the sensory profile of the species under investigation and their consumer acceptability, HI results closer to those of wild species were obtained in species domesticated using the OF method, usually followed by domestication in pots, except for *R. acetosa* and *P: coronopus.* On the contrary, the most different sensory profile compared with that of the wild species was attributed to *P. hieracioides* and *C. intybus* domesticated using the SS method.

## 5. Conclusions

The analysis of the sensory and phytochemical characterization of five wild edible herbs, with the aim of identifying their potential nutraceutical properties and ascertaining the range of palatability through the sensory profile, unveiled the wild-collected plants as promising for “new” functional food. Three completely different attempts of domestication were evaluated in this study.

Leaves of plants domesticated with the OF method had nutraceutical and sensory profiles similar to those of the wild species, especially in *C. intybus*, *P. hieracioides*, and *S. minor.* Leaves of plants domesticated with P method showed a pigment content close to that of the wild species, as well as a lower phytochemical content than that of the wild species.

In the SS method, *R. acetosa* and *P. coronopus* displayed a high quality in terms of phytochemicals and antioxidant activity. Therefore, we can conclude that different species respond differently to different domestications and that the use of domestication in open air is more appropriate for herbs that naturally grow in the wild. However, greenhouse domestications cannot be excluded in terms of phytochemicals and nutraceutical properties, dependent on the species.

Finally, in terms of the preliminary assessment carried out in this study through the analysis of general nutraceutical properties, *S. minor* exhibited intrinsically higher nutraceutical properties than the other examined species, as well as common vegetables, independent of domestication or wild harvest methods. However, further research on the bioavailability and bioactivity tests of this species is required to confirm the findings of this study. Moreover, further research may also be useful to consider the storage of freshly cut *S. minor* and to study the effective marketability and conservation of this species as a “new” functional food.

## Figures and Tables

**Table 1:** Two-way ANOVA calculated for all quantitative and hedonic parameters evaluated by panelists during tasting sessions for *Rumex acetosa*. Only data that showed significant differences (*p* < 0.05) are reported. Means keyed with a different letter are significantly different for *p* = 0.05, considering each species independently and following one-way ANOVA, with wild-collection or the different domestications as the variability factor. Significance level: ***: *p* < 0.001 (F = 7.94); **: *p* < 0.01 (F = 4.77); *: *p* < 0.05 (F = 3.01).

Quantitative Parameter	*p*-Value	W	SS	P	OF
**View Attributes**					
Homogeneity of dimensions	**	4.9a	2.8b	2.4b	3.6ab
Homogeneity of shape	**	6.40a	5.8ab	4.2b	4.0b
Color intensity	***	7.1a	5.8b	5.2b	5.4b
Hue (yellow/green)	***	7.3a	6.9a	4.7b	5.4b
Leaf fuzzy on underside	***	1.4a	0b	0b	0b
**Smell Attributes**					
Odor intensity	***	7.5a	3.6c	5.6b	6.8ab
**Touch/Rheological Sensations**					
Smooth	***	5.8b	8.0a	7.0a	8.0a
Wrinkled	***	3.8a	2.0ab	1.6bc	0c
Flabbiness	***	2.6a	1.0ab	4.0bc	2.2c
Rubbery	***	0b	0.4b	4.2a	1.4b
Resistance to chewing	**	4.0b	7.8a	4.2b	3.0b
**Taste Attributes/Trigeminal Sensations**					
Acid	**	7.8a	7.2ab	8.0a	6.2b
Hot	***	0b	1.6a	0b	0b
Intensity of taste	***	8.0a	8.0a	7.8a	4.8b
Complexity of taste	**	2.1b	4.6ab	5.0a	3.4ab
Juiciness	**	3.6b	5.8ab	6.6a	6.5a
Astringency	*	2.1ab	0.4b	4.2a	1.4ab
**Hedonic Parameter**	***p*-Value**	**W**	**SS**	**P**	**OF**
Mouthfeel pleasantness	**	4.5a	2.6b	3.7ab	2.9ab

**Table 2:** Two-way ANOVA calculated for all quantitative and hedonic parameters evaluated by panelists during tasting sessions for *Cichorium intybus.* Only data that showed significant differences (*p* < 0.05) are reported. Means keyed with a different letter are significantly different for *p* = 0.05, considering each species independently and following one-way ANOVA, with wild-collection or the different domestications as the variability factor. Significance level: ***: *p* < 0.001 (F = 7.94); **: *p* < 0.01 (F = 4.77); *: *p* < 0.05 (F = 3.01).

Quantitative Parameter	*p*-Value	W	SS	P	OF
**View attributes**					
Homogeneity of dimensions	***	5.8a	3.2b	3.6b	2.4b
Homogeneity of shape	**	5.6ab	8.0a	5.0b	4.0b
Color intensity	***	6.6c	9.0a	7.9b	8.0b
Color regularity	***	7.2a	5.1b	6.0b	7.6a
Leaf fuzzy on underside	***	0.6b	0b	3.6a	0.6b
**Smell attributes**					
Odor intensity	***	7.4a	7.9a	2.6c	5.6b
**Touch/Rheological sensations**					
Smooth	***	7.8a	8.0a	3.4b	8.0a
Wrinkled	***	0b	1.0b	4.8a	0b
Pungent	*	0b	0b	1.2a	0b
Flabbiness	***	4.7b	6.2a	4.3b	4.7b
Woody	***	2.1a	0b	0b	0b
Resistance to chewing	***	6.6a	2.4b	6.6a	7.2a
**Taste attributes/Trigeminal sensations**					
Acid	***	2.0b	2.0b	5.0a	2.8b
Salty	***	3.2a	0b	4.4a	3.9a
Intensity of taste	**	7.4ab	6.9b	8.2a	6.8b
Complexity of taste	**	1.4b	3.0ab	3.4ab	4.4a
Juiciness	***	0.8c	2.3bc	2.8b	5.8a
Aftertaste	*	0.4ab	0b	2.2a	1.4ab
**Hedonic Parameter**	***p*-value**	**W**	**SS**	**P**	**OF**
Visual attractiveness	***	7.9a	4.0c	6.8b	8.0a
Mouthfeel pleasantness	**	3.3b	4.2ab	4.8a	5.3a
Persistency	**	4.4ab	3.0b	5.7a	6.6a

**Table 3:** Two-way ANOVA calculated for all quantitative and hedonic parameters evaluated by panelists during tasting sessions for *Picris hieracioides*. Only data that showed significant differences (*p* < 0.05) are reported. Means keyed with a different letter are significantly different for *p* = 0.05, considering each species independently and following one-way ANOVA, with wild-collection or the different domestications as the variability factor. Significance level: ***: *p* < 0.001 (F = 7.94); **: *p* < 0.01 (F = 4.77); *: *p* < 0.05 (F = 3.01).

Quantitative Parameter	*p*-Value	W	SS	P	OF
**View attributes**					
Homogeneity of dimensions	***	5,2bc	6.4ab	3.3c	7.4a
Homogeneity of shape	***	5.2b	8.0a	6.4ab	7.4a
Color intensity	**	8.1a	6.5b	6.9b	7.1ab
Color regularity	***	8.1a	4.0c	4.8bc	6.6ab
Hue (yellow/green)	***	8.3a	7.0b	7.2b	7.0b
Leaf fuzzy on upper side	***	7.8bc	7.0c	9.0a	8.9ab
Leaf fuzzy on underside	***	7.9b	6.0c	9.0a	8.8a
**Touch/Rheological sensations**					
Wrinkled	**	04ab	2.0b	6.2a	3.8ab
Pungent	***	8.8a	7.0b	9.0a	9.0a
Flabbiness	***	0b	0b	0b	4.4a
Woody	***	2.6b	0c	7.6a	3.8b
Resistance to chewing	**	5.4b	8.6a	5.4b	6.8ab
**Taste attributes/Trigeminal sensations**					
Acid	***	4.0a	0.8b	5.6a	3.6a
Salty	**	4.2a	1.0b	4.6a	3.8ab
Bitter	***	9.0a	9.0a	7.0b	8.8a
Hot	*	0b	0b	0.4ab	1.8a
Intensity of taste	***	8.4ab	9.0a	6.0c	7.6b
Complexity of taste	***	1.2b	0.8b	4.2a	4.8a
Juiciness	***	1.2b	1.0b	3.8a	5.2a
Astringency	***	0.2b	0b	5.0a	3.4a
**Hedonic Parameter**	***p*-value**	**W**	**SS**	**P**	**OF**
Visual attractiveness	***	6.6a	2.7b	3.2b	3.0b
Mouthfeel pleasantness	***	3.6bc	1.0c	3.8b	6.8a
Persistency	***	3.3b	4.2b	4.9b	8.5a

**Table 4:** Two-way ANOVA calculated for all quantitative and hedonic parameters evaluated by panelists during tasting sessions for *Sanguisorba minor*. Only data that showed significant differences (*p* < 0.05) are reported. Means keyed with a different letter are significantly different for *p* = 0.05, considering each species independently and following one-way ANOVA, with wild-collection or the different domestications as the variability factor. Significance level: ***: *p* < 0.001 (F = 7.94); **: *p* < 0.01 (F = 4.77); *: *p* < 0.05 (F = 3.01).

Quantitative Parameter	*p*-Value	W	SS	P	OF
**View Attributes**					
Homogeneity of dimensions	***	7.3a	6.4a	2.6b	6.8a
Color intensity	*	7.8a	7.2ab	7.0ab	6.5b
**Smell Attributes**					
Odor intensity	**	5.3b	5.0b	6.4ab	7.8a
**Touch/Rheological Sensations**					
Smooth	***	2.4c	5.4b	5.6ab	8.0a
Wrinkled	***	0.8b	5.0a	4.4a	0b
Pungent	***	4.4a	0b	0b	0b
Woody	***	0b	3.0a	2.0a	2.6a
Resistance to chewing	***	1.6c	8.0a	2.4bc	4.0b
**Taste Attributes/Trigeminal Sensations**					
Sweet	***	0b	0b	1.8a	1.2ab
Bitter	***	7.2a	4.8ab	3.2bc	2.2c
Intensity of taste	*	5.8a	5.0ab	5.2ab	3.2b
Complexity of taste	***	2.6b	8.0a	6.2a	2.8b
Astringency	**	7.0a	6.0a	5.0ab	3.0b
**Hedonic Parameter**	***p*-Value**	**W**	**SS**	**P**	**OF**
Visual attractiveness	**	8.1ab	9.0a	7.6b	7.1b
Overall pleasantness	*	3.0ab	2.6b	4.5ab	4.6a
Persistency	***	2.6c	4.0b	5.7a	3.6bc

**Table 5:** Two-way ANOVA calculated for all quantitative and hedonic parameters evaluated by panelists during tasting sessions for *Plantago coronopus*. Only data that showed significant differences (*p* < 0.05) are reported. Means keyed with a different letter are significantly different for *p* = 0.05, considering each species independently and following one-way ANOVA, with wild-collection or the different domestications as the variability factor. Significance level: ***: *p* < 0.001 (F = 7.94); **: *p* < 0.01 (F = 4.77); *: *p* < 0.05 (F = 3.01).

Quantitative Parameter	*p*-Value	W	SS	P	OF
**View Attributes**					
Homogeneity of dimensions	***	7.2a	6.0ab	4.4b	7.8a
Homogeneity of shape	***	7.1a	5.6ab	3.0b	4.4b
Color intensity	***	7.1a	4.2ab	5.7b	6.7a
Hue (yellow/green)	***	7.4a	6.0b	6.2b	6.6ab
Leaf fuzzy on underside	**	3.0a	0.6b	3.0a	0.5b
**Touch/Rheological Sensations**					
Smooth	***	0.2c	7.9a	5.6b	7.8a
Wrinkled	*	5.0a	0.2b	3.2ab	1.6ab
Pungent	***	2.0a	0b	0.4b	0b
Woody	***	5.8a	0b	4.6a	0b
Resistance to chewing	**	8.9a	7.8ab	5.2b	6.0b
**Taste Attributes/Trigeminal Sensations**					
Sweet	***	1.0b	2.6a	2.0a	2.6a
Acid	**	2.8ab	4.3a	4,60a	2.2b
Bitter	***	2.1a	0.4b	3.0a	0.4b
Intensity of taste	**	4.2b	4.0b	6.2a	5.9ab
Complexity of taste	***	3.4b	4.5b	7.4a	5.0ab
Juiciness	*	3.0b	4.4ab	6.0ab	7.0a
**Hedonic Parameter**	***p*-Value**	**W**	**SS**	**P**	**OF**
Mouthfeel pleasantness	**	7.4a	5.1b	6.2ab	5.0b
Overall pleasantness	***	5.2b	7.9a	7.3a	7.6a
Persistency	***	6.2a	4.5b	4.2b	6.7a

## Supplementary Materials

The following are available online at https://www.mdpi.com/2304-8158/9/8/1065/s1, Table S1. Details of the three domestication methods and of the wild harvest. Table S2. Pearson’s correlation coefficient among quantitative and hedonic parameters. Strong correlations were highlighted in grey.
