# Supplementary material for: Comparison of Three Domestications and Wild-Harvested Plants for Nutraceutical Properties and Sensory Profiles in Five Wild Edible Herbs: Is Domestication Possible?
Source: Foods. 2020 Aug 6;9(8):1065. doi: 10.3390/foods9081065 (PMC7466383; doi:10.3390/foods9081065)
Supplement: Supplementary file 1 [file foods-09-01065-s001.docx]

| **Domestication method** | **Seeds** | **Growing Area/Location** | **Number of seedlings for each examined species** | **Duration Growing Cycle** | **Soil/Substrate** | **Pre-treatment of soil/Fertirrigation** | **Average Temperature** | **Average Relative Humidity** | **Daily Mean Solar Irradiation** |
| --- | --- | --- | --- | --- | --- | --- | --- | --- | --- |
| W | Wild | Pisa, Italy | 10 | One month^1^ | Mainly sandy soil (21% clay, 49% sand, 30% silt, 2% organic matter) | None | 21.3 °C | 57.5% | 1050 W m^–2^ |
| SS | Gargini Sementi S.n.c. (Lucca, Italy) | Greenhouse  Pisa, Italy | 10 | 35 days^1^ | Peat | Nutritive solution^2^ | 15.9 °C | 79.6% | 287.5 W m^–2^ |
| P | Gargini Sementi S.n.c. (Lucca, Italy) | Greenhouse  Siena, Italy | 10 | 36 days^1^ | Peat | Nutritive solution^2^ | 23.9 °C | 52.1% | 365 W m^–2^ |
| OF | Gargini Sementi S.n.c. (Lucca, Italy) | Siena, Italy | 10 | One month^1^ | Mainly silt soil (31% clay, 24% sand, 45% silt, 2% organic matter). | Manure (30 kg ha–1) as pre-treatment of soil, | 24.5 °C | 46.0% | 1050 W m^–2^ |

**Table S1**. Details of the three domestication methods and of the wild harvest.

^1^Necessary period for the growth of young aerial parts, adapted to the human consumption. ^2^The nutritive solution used as fertigation in SS and P domestication were composed of NO_3_^–^ 10 mM, NH_4_^+^ 0.5 mM, PO_4_^3–^ 1 mM, K^+^ 6 mM, Ca^2+^ 4 mM, Mg^2+^ 2 mM, Na^+^ 0.5 mM, SO_4_^2–^ 3.5 mM, Cl^–^ 0.5 mM, HCO_3_^–^ 0.5 mM, Fe^2+^ 40 µM, BO_3_^–^ 25 µM, Cu^2+^ 1 µM, Zn^2+^ 10 µM and Mo^3+^ 1 µM

**Table S2.** Pearson’s correlation coefficient among quantitative and hedonic parameters. Strong correlations were highlighted in grey.

| ***Rumex acetosa*** | | | | | |
| --- | --- | --- | --- | --- | --- |
|  | **Visual attractiveness** | **Mouthfeel pleasantness** | | **Persistency** | **Overall pleasantness** |
| **Homogeneity of dimensions** | 0.55 | | nd^1^ | nd | 0.02 |
| **Homogeneity of shape** | 0.09 | | nd | nd | –0.21 |
| **Colour intensity** | 0.14 | | nd | nd | –0.25 |
| **Colour regularity** | 0.15 | | nd | nd | 0.13 |
| **Hue (yellow/green)** | 0.12 | | nd | nd | –0.20 |
| **Leaf fuzzy on upper sidee** | 0.32 | | nd | nd | 0.04 |
| **Leaf fuzzy on under side** | –0.31 | | nd | nd | –0.56 |
| **Odour intensity** | nd | | 044 | –0.45 | –0.25 |
| **Smooth** | nd | | –0.47 | 0.34 | 0.47 |
| **Wrinkled** | nd | | 0.61 | –0.30 | –0.41 |
| **Pungent** | nd | | 0.00 | 0.,00 | 0.00 |
| **Flabbiness** | nd | | 0.30 | –0.06 | –0.26 |
| **Rubbery** | nd | | 0.01 | 0.12 | 0.21 |
| **Woody** | nd | | 0.24 | –0.35 | 0.01 |
| **Resistance to chewing** | nd | | –0.46 | 0.05 | 0.19 |
| **Sweet** | nd | | –0.31 | 0.15 | 0.10 |
| **Acid** | nd | | 0.21 | 0.07 | –0.44 |
| **Salty** | nd | | 0.19 | –0.26 | –0.51 |
| **Bitter** | nd | | 0.14 | –0.16 | –0.10 |
| **Hot** | nd | | –0.56 | 0.09 | 0.11 |
| **Intensity of taste** | nd | | 0.13 | –0.03 | –0.33 |
| **Complexity of taste** | nd | | –0.34 | 0.41 | 0.50 |
| **Juiciness** | nd | | –0.49 | 0.34 | 0.44 |
| **Astringency** | nd | | 0.03 | –0.23 | 0.20 |
| **Aftertaste** | nd | | 0.05 | 0.13 | 0.10 |
| ***Cichorium intybus*** | | | | | |
|  | **Visual attractiveness** | | **Mouthfeel pleasantness** | **Persistency** | **Overall pleasantness** |
| **Homogeneity of dimensions** | 0.29 | | nd | nd | –0.32 |
| **Homogeneity of shape** | –0.51 | | nd | nd | 0.06 |
| **Colour intensity** | –0.63 | | nd | nd | 0.21 |
| **Colour regularity** | 0.77 | | nd | nd | 0.23 |
| **Hue (yellow/green)** | –0.47 | | nd | nd | 0.14 |
| **Leaf fuzzy on upper side** | 0.25 | | nd | nd | 0.01 |
| **Leaf fuzzy on under side** | 0.04 | | nd | nd | –0.12 |
| **Odour intensity** | nd | | –0.52 | –0.52 | –0.04 |
| **Smooth** | nd | | –0.22 | –0.43 | 0.10 |
| **Wrinkled** | nd | | 0.33 | 0.29 | 0.04 |
| **Pungent** | nd | | 0.22 | 0.34 | –0.31 |
| **Flabbiness** | nd | | –0.01 | –0.24 | –0.03 |
| **Rubbery** | nd | | 0.54 | –0.01 | –0.20 |
| **Woody** | nd | | –0.71 | –0.21 | –0.23 |
| **Resistance to chewing** | nd | | 0.14 | 0.45 | 0.04 |
| **Sweet** | nd | | 0.00 | 0.00 | 0.00 |
| **Acid** | nd | | 0.33 | 0.41 | –0.13 |
| **Salty** | nd | | 0.10 | 0.54 | –0.06 |
| **Bitter** | nd | | 0.15 | –0.10 | 0.28 |
| **Hot** | nd | | 0.00 | 0.00 | 0.00 |
| **Intensity of taste** | nd | | –0.13 | 0.08 | –0.15 |
| **Complexity of taste** | nd | | 0.84 | 0.03 | 0.29 |
| **Juiciness** | nd | | 0.63 | 0.40 | 0.54 |
| **Astringency** | nd | | 0.20 | 0.38 | –0.36 |
| **Aftertaste** | nd | | 0.47 | 0.23 | 0.25 |
| ***Picris hieracioides*** | | | | | |
|  | **Visual attractiveness** | | **Mouthfeel pleasantness** | **Persistency** | **Overall pleasantness** |
| **Homogeneity of dimensions** | –0.11 | | nd | nd | –0.31 |
| **Homogeneity of shape** | –0.58 | | nd | nd | –0.20 |
| **Colour intensity** | 0.61 | | nd | nd | 0.24 |
| **Colour regularity** | 0.72 | | nd | nd | 0.06 |
| **Hue (yellow/green)** | 0.79 | | nd | nd | 0.23 |
| **Leaf fuzzy on upper side** | –0.11 | | nd | nd | 0.14 |
| **Leaf fuzzy on under side** | 0.10 | | nd | nd | 0.22 |
| **Odour intensity** | nd | | 0.24 | 0.39 | 0.28 |
| **Smooth** | nd | | 0.00 | 0.00 | 0.00 |
| **Wrinkled** | nd | | 0.25 | 0.42 | –0.10 |
| **Pungent** | nd | | 0.51 | 0.18 | 0.20 |
| **Flabbiness** | nd | | 0.67 | 0.80 | –0.27 |
| **Rubbery** | nd | | 0.00 | 0.00 | 0.00 |
| **Woody** | nd | | 0.39 | 0.15 | 0.12 |
| **Resistance to chewing** | nd | | –0.15 | 0.16 | 0.10 |
| **Sweet** | nd | | –0.16 | 0.01 | 0.44 |
| **Acid** | nd | | 0.54 | –0.07 | 0.39 |
| **Salty** | nd | | 0.62 | 0.07 | 0.09 |
| **Bitter** | nd | | –0.17 | –0.07 | –0.52 |
| **Hot** | nd | | 0.58 | 0.64 | –0.05 |
| **Intensity of taste** | nd | | –0.35 | –0.12 | –0.34 |
| **Complexity of taste** | nd | | 0.63 | 0.63 | –0.09 |
| **Juiciness** | nd | | 0.65 | 0.72 | –0.13 |
| **Astringency** | nd | | 0.47 | 0.48 | 0.23 |
| **Aftertaste** | nd | | 0.02 | 0.42 | –0.17 |
| ***Sanguisorba minor*** | | | | | |
|  | **Visual attractiveness** | | **Mouthfeel pleasantness** | **Persistency** | **Overall pleasantness** |
| **Homogeneity of dimensions** | 0.29 | | nd | nd | –0.33 |
| **Homogeneity of shape** | 0.22 | | nd | nd | –0.14 |
| **Colour intensity** | 0.34 | | nd | nd | –0.21 |
| **Colour regularity** | 0.35 | | nd | nd | –0.46 |
| **Hue (yellow/green)** | 0.50 | | nd | nd | 0.01 |
| **Leaf fuzzy on upper side** | 0.00 | | nd | nd | 0.00 |
| **Leaf fuzzy on under side** | 0.11 | | nd | nd | –0.34 |
| **Odour intensity** | nd | | 0.61 | 0.12 | 0.42 |
| **Smooth** | nd | | 0.39 | 0.30 | 0.47 |
| **Wrinkled** | nd | | –0.13 | 0.68 | –0.24 |
| **Pungent** | nd | | –0.28 | –0.65 | –0.24 |
| **Flabbiness** | nd | | 0.21 | –0.06 | 0.14 |
| **Rubbery** | nd | | 0.41 | 0.09 | 0.10 |
| **Woody** | nd | | 0.44 | 0.40 | 0.36 |
| **Resistance to chewing** | nd | | 0.10 | 0.09 | –0.10 |
| **Sweet** | nd | | 0.17 | 0.46 | 0.39 |
| **Acid** | nd | | 0.08 | –0.08 | 0.11 |
| **Salty** | nd | | 0.32 | 0.16 | 0.47 |
| **Bitter** | nd | | –0.17 | –0.64 | –0.21 |
| **Hot** | nd | | 0.30 | 0.37 | 0.22 |
| **Intensity of taste** | nd | | 0.10 | –0.08 | –0.51 |
| **Complexity of taste** | nd | | 0.03 | 0.48 | –0.13 |
| **Juiciness** | nd | | 0.54 | –0.10 | 0.39 |
| **Astringency** | nd | | –0.41 | –0.42 | –0.23 |
| **Aftertaste** | nd | | 0.24 | 0.21 | 0.27 |
| ***Plantago coronopus*** | | | | | |
|  | **Visual attractiveness** | | **Mouthfeel pleasantness** | **Persistency** | **Overall pleasantness** |
| **Homogeneity of dimensions** | 0.67 | | nd | nd | –0.15 |
| **Homogeneity of shape** | 0.65 | | nd | nd | –0.38 |
| **Colour intensity** | 0.53 | | nd | nd | –0.49 |
| **Colour regularity** | –0.25 | | nd | nd | 0.22 |
| **Hue (yellow/green)** | 0.65 | | nd | nd | –0.69 |
| **Leaf fuzzy on upper side** | 0.21 | | nd | nd | –0.31 |
| **Leaf fuzzy on under side** | 0.31 | | nd | nd | –0.44 |
| **Odour intensity** | nd | | –0.14 | 0.03 | 0.23 |
| **Smooth** | nd | | –0.64 | –0.27 | 0.81 |
| **Wrinkled** | nd | | 0.52 | 0.17 | –0.74 |
| **Pungent** | nd | | 0.68 | 0.36 | –0.71 |
| **Flabbiness** | nd | | –0.25 | –0.34 | 0.69 |
| **Rubbery** | nd | | –0.10 | –0.45 | 0.43 |
| **Woody** | nd | | 0.72 | 0.06 | –0.61 |
| **Resistance to chewing** | nd | | 0.37 | 0.20 | –0.42 |
| **Sweet** | nd | | –0.24 | –0.01 | 0.85 |
| **Acid** | nd | | –0.24 | –0.53 | 0.22 |
| **Salty** | nd | | –0.06 | 0.04 | 0.44 |
| **Bitter** | nd | | 0.31 | –0.14 | –0.33 |
| **Hot** | nd | | 0.11 | –0.09 | 0.19 |
| **Intensity of taste** | nd | | –0.24 | 0.09 | 0.37 |
| **Complexity of taste** | nd | | –0.08 | –0.19 | 0.64 |
| **Juiciness** | nd | | –0.15 | 0.08 | 0.58 |
| **Astringency** | nd | | 0.21 | –0.02 | –0.37 |
| **Aftertaste** | nd | | 0.12 | –0.19 | –0.24 |

^1^ nd: not detected
